# Multifamily therapy for adolescent eating disorders: a study of the change in eating disorder symptoms from start of treatment to follow-up

**DOI:** 10.1186/s40337-023-00814-y

**Published:** 2023-06-07

**Authors:** Ingrid Funderud, Inger Halvorsen, Anne-Lise Kvakland, Jan-Vegard Nilsen, Jeanette Skjønhaug, Kristin Stedal, Øyvind Rø

**Affiliations:** 1 Regional Department for Eating Disorders, Division of Mental Health and Addiction, Oslo University Hospital, Oslo, Norway; 2 Division of Mental Health and Addiction, Institute of Clinical Medicine, University of Oslo, Oslo, Norway

**Keywords:** Eating disorders, Adolescents, Multifamily therapy, Outcome

## Abstract

**Background:**

This study aimed to evaluate multifamily therapy (MFT) for adolescents with eating disorders (EDs) in a clinical setting, by presenting the outcome of families participating in this treatment at a specialist ED service. MFT was an adjunct to treatment at local mental health services. In particular, the study aimed to present the change in eating disorder symptoms and psychological distress from before to after treatment and at a 6 months follow-up.

**Methods:**

Participants were 207 adolescents receiving outpatient MFT (10 or 5 months) at Oslo University Hospital in Norway between 2009 and 2022. Adolescents had heterogeneous ED presentations, with a preponderance of anorexia nervosa (AN) and atypical AN. All participants completed pre- and post-treatment questionnaires [The eating disorder examination questionnaire (EDE-Q) and the strengths and difficulties questionnaire (SDQ)]. 142 adolescents additionally completed the same questionnaires at 6 months follow-up. Weight and height were measured at all time points.

**Results:**

Linear mixed model analyses showed that from start of treatment to follow-up, there was a significant increase in BMI percentile (*p* < 0.001) and a significant decrease in EDE-Q global score (*p* < 0.001) and SDQ total score (*p* < 0.001).

**Conclusions:**

The study shows that adolescents with an eating disorder who received adjunct outpatient MFT in a real world clinical setting, experienced reductions in ED symptoms comparable to that found in a randomized controlled trial.

*Trial registration*: The data used in this study was collected as part of routine clinical procedures for quality assurance and trial registration is therefore not required.

## Abstract (plain language summary)

Eating disorders are serious psychiatric diseases associated with a significant disease burden. Providing effective treatment is imperative. In multifamily therapy, several families are brought together in multi-family groups to work together to overcome the eating disorder. Research on multifamily therapy suggests that it is effective in treating adolescent eating disorder, but there is a need for studies with larger sample sizes to evaluate how the treatment performs outside controlled research settings. Data were collected from 207 adolescents participating in multifamily therapy at a specialist eating disorder service in Norway. There were significant improvements in eating disorder symptoms and psychological distress from start of treatment to 6 month follow-up. The study shows that adolescents with an eating disorder who received multifamily therapy in a real world clinical setting, experienced reductions in eating disorder symptoms comparable to that found in a randomized controlled trial and in smaller studies from real world settings.

## Background

Multifamily therapy for eating disorders (MFT) was developed with the intention to modify and intensify single-family therapy. In MFT several families are brought together in multi-family groups to work together to overcome the eating disorder (ED). The first MFT programs for eating disorders were introduced in the 1990’s [1, 2]. These programs, and later versions of the programs (e.g., MFT-AN, [3]), draw heavily on single-family therapy (e.g. family-based treatment (FBT; [4]) and the Maudsley model of family therapy (FT–AN; [5]) and are delivered adjunct to single-family therapy. At present, many MFT programs exist. They all have in common the concept that families facing similar problems can share experiences, and support and learn from each other [6, 7]. Hearing how other families deal with similar problems, and discussing with the different members of the other families, initiates implicit learning. It helps the families creating new perspectives and gives the possibility for mutual support and feedback [8, 9]. Meeting other families in the same situation also helps adolescents and their family members to overcome the isolation and stigmatization that often comes with having an ED or an eating disordered family member [2, 10]. Despite common factors, there is great variability in the way MFT is delivered in terms of setting, recruitment, treatment intensity, treatment duration, and number of families taking part in each MFT group [11, 12]. For example, MFT is delivered as outpatient treatment, inpatient treatment, and as a day program, and manuals have been developed for children and adolescents as well as for adults, and for anorexia nervosa (AN) and atypical AN (e.g., [3, 13]) as well as for Bulimia Nervosa [14]. Although MFT is typically delivered as adjunct treatment (e.g., [2, 3, 13]), it is also offered as stand-alone treatment [15, 16].

MFT is now widely used, and is a recommended treatment for adolescents by several guidelines [17, 18], but research evidence is still scarce. A recent review [19] identified 27 studies on MFT (17 quantitative, of which 10 reported on outpatient MFT for young people (i.e., under 25 years)). The review revealed promising results for MFT, including improvements in ED psychopathology, weight, global outcome, and depression. However, most of the included studies had small sample sizes, and for young people, only two of the studies included more than hundred participants [9, 20], while the other studies had samples consisting of between 15 and 82 participants. The studies on adult patients included even fewer participants, with a maximum of 68 [21]. A more recent study by Terache and colleagues (2022) also found significant improvements in ED psychopathology and weight increase. The improvements were maintained at 6- and 12-month follow-ups [22]. Thus, the somewhat limited literature of outpatient MFT for adolescents shows improvements in ED symptomatology, weight, depression, and global outcome after MFT, with some benefit of MFT over other treatment. However, several authors have pointed out the challenges with implementing evidence-based treatments in real world clinics [23–25]. To evaluate the effectiveness of evidence-based treatments outside a controlled research setting, there is a need for naturalistic studies on the outcome of the treatment in real world clinical settings and at sites beyond those of the primary research. In their review, Baudinet et al. (2021) concluded that it is crucial to gain knowledge on the effectiveness of MFT treatment outside of research settings and that there is a need for studies with larger sample sizes [19].

The main aim of this naturalistic study was to report the outcome of adjunct MFT for adolescents with eating disorders in a real world clinical setting. With this aim, we investigated changes in clinical outcomes (e.g. body weight, self-reported ED symptoms and psychological problems) from start of MFT to end of MFT (hereafter start-of-treatment and end-of-treatment) and to a 6-month follow-up at a real world specialist ED clinic.

## Methods

### Participants

Participants were adolescents (11–21 years of age, 91% female) seen for outpatient multifamily treatment at Regional Department for Eating Disorders (RASP), Oslo University Hospital in Norway between 2010 and 2021. RASP is a specialist ED service for children, adolescents and adults in Southern and Eastern Norway. Patients were referred from a large number of local specialized mental health services.

The current investigation includes data from patients treated with outpatient MFT at RASP from May 2010 to March 2022 (224 patients). Seventeen patients did not respond to any questionnaires, and were therefore excluded from the analyses. The final dataset thus consisted of data from 207 patients. From 2015 onwards the adolescents and their families were seen for follow-up (after 6 months), thus the dataset includes follow-up data from 142 adolescents.

International Classification of Diseases, 10th edition (ICD-10) [26] diagnoses were established by an experienced clinician. Structural diagnostic interviews were not performed. The diagnoses at start of MFT were based on clinical information on height, weight, BMI-percentile, weight loss, menstrual cycle, eating (disordered) behavior, and information from the referral regarding psychological and cognitive ED related disturbances. For this study, ICD-10 diagnoses F50.9 and F50.8 were coded together as “Unspecified or other eating disorder”.

At start of MFT 92.7% of the patients met the criteria for an AN diagnosis (N = 164; 78 F.50.0 AN and 86 F50.1 Atypical AN), 6.8% met the criteria for a Bulimia Nervosa (BN) diagnosis (N = 5; 4 F50.2 BN and 1 F50.3 Atypical BN), and 4.5% (N = 8) had a diagnosis of F50.9/F50.8 Unspecified or other eating disorder. For 30 of the 207 patients we did not have sufficient information to make a diagnostic evaluation at start-of-treatment. Many of the patients with F50.1 at start of MFT had previously fulfilled criteria for F50.0, but because of weight gain during the treatment at local mental health services, they did not fulfill the weight criterion for the F50.0-diagnosis at start of MFT.

### Missing data

One hundred and thirty adolescents responded to questionnaires at all eligible time points (i.e. two time points prior to 2015, and three time points after 2015), 160 patients responded to questionnaires at two time-points, and 44 responded to questionnaires at a single time point only (37 at start-of-treatment, 5 at end-of-treatment, and 2 at follow-up). Comparing adolescents who had questionnaire data at start-of-treatment only to adolescents with complete datasets, there were no differences in age, gender, ED diagnosis, BMI percentile at start-of-treatment, SDQ total score, EDE-Q global score, or age at first treatment (*p*’s > 0.219).

We had weight and height at all time points for 127 patients and at start-of-treatment and end-of-treatment for 159 patients. For 45 patients, weight and height data were collected at start-of treatment only.

### Intervention

The MFT treatment provided at RASP is delivered according to the Multi-family therapy for adolescent anorexia nervosa manual (MFT-AN) [1, 3] and is described in detail in the treatment manual [27] written by U. Wallin after receiving MFT training at Maudsley Centre for Child and Adolescent Eating Disorders.

At RASP MFT is offered to adolescent patients and their families as an adjunct to ED treatment provided at each patient’s local mental health services (mostly 1 session/week at the start of MFT, while fewer by the end of MFT). This is in accordance with the intention that MFT-AN interventions should intensify and modify single family therapy for ED [3]. Some of the local mental health services provided FBT or other forms of single family therapy during the whole period covered by the current study, while others did not provide FBT until 2017. In the national guidelines published in 2017 [28] FBT was recommended as treatment for children and adolescents with ED, and thereafter FBT has over the course of several years been implemented as the main treatment for adolescent ED at the local specialized mental health services. RASP has a catchment area with 3 million inhabitants, constituting about half of Norway’s population.

The treatment consisted of 10–11 full days of treatment. Initially the 10 days of treatment were spread across approximately 10 months, but in 2015, treatment duration was shortened to 5 months because many families expressed that a duration of 10 months was too long. The number of treatment days remained unchanged. Thus, for 65 of the patients included in this study, the treatment lasted for a mean of 10.2 months (SD: 1.2, range 6–13), and for 142 patients it lasted for a mean of 5.1 months (SD: 1.1, range 4–7).

The groups were made up of between four and eleven patients and their families (mean group size: 6 families, SD: 1.8) and were led by an experienced multidisciplinary treatment team consisting of family therapists, psychiatric nurses, medical doctors and psychologists. The treatment team had 4 to 6 members; two lead therapists and up to four facilitators. The lead therapists were experienced family therapists who ran the MFT groups on a regular basis. The facilitators included other staff from RASP who joined MFT groups occasionally. They all had extensive clinical experience with eating disorders. Facilitators also often included up to two trainees who were clinicians from specialized mental health services who wanted to learn about MFT. The lead therapists were trained in MFT by Maudsley Centre for Child and Adolescent Eating Disorders, London, UK and at Lund University, Sweden. To ensure fidelity to the model, therapists from Maudsley have visited the Scandinavian MFT network to provide additional teaching and supervision.

The MFT starts with a four-day intensive workshop where the focus is on illness-related themes (managing meals, impact of illness on family life etc.). Across the remaining days, themes are progressively moving away from illness related themes to broader adolescent and family lifecycle challenges. The treatment involves plenum sessions, and group work in smaller groups (e.g. separate groups for patients, siblings, mothers and fathers, mixed groups where people from different families work together, within-family groups). The MFT provided at RASP has only a very few adaptations from the MFT-AN manual. Instead of meeting six single days after the initial four-day intensive workshop, the group meets two days in a row three times, with an additional single day at the end for some groups. The MFT-AN manual suggests that the families have three meals together as a large group. At RASP, only lunch is eaten with all the families together. The therapists also observe and coach the families during this meal to a lesser extent than described in the manual. Moreover, the food is provided by RASP instead of the families bringing their own food. Another adaptation is that in addition to the manualized exercises and group discussions, two lectures are given; one about self-esteem, and one about the neurobiology of eating disorders. Lastly, cultural adaptations are made, by choosing activities that are most suited to a Norwegian population [28].

### Measures

#### Self-report questionnaires

*The EDE Questionnaire* 6.0 (EDE-Q) [29] assessed core ED symptoms and behaviors during the previous 28 days. The EDE-Q has four subscales (dietary restraint, eating concern, weight concern and shape concern) which are used to calculate the global score. Global score ranges from 0 to 6, with higher scores reflecting greater pathology. The global score was used in this study as a measure of eating disorder symptomology severity. An EDE-Q global score of ≤ 2.5 has been demonstrated as the clinical threshold to optimally discriminate between controls and ED patients in a sample of Norwegian women [30]. Therefore, 2.5 was considered as an appropriate cut-off core for the sample of the current study. The Norwegian version of EDE-Q has shown satisfactory psychometric properties [30–32].

*The Strengths and Difficulties Questionnaire* (SDQ) [33] measured self-rated behavioral and psychological problems during the previous 6 months. The questionnaire comprises 25 items divided into 5 subscales (emotional problems, conduct problems, hyperactivity and peer problems, prosocial). A total difficulties score is generated by summing scores from all the subscales except the prosocial subscale, and ranges from 0 to 40 with higher score indicating more difficulties. SDQ has good psychometric properties [34–36].

The questionnaires were administered at start-of-treatment, end-of-treatment and follow-up.

#### Weight and height

To calculate Body Mass Index (BMI) percentile weight and height was recorded by clinicians at start-of-treatment, end-of-treatment, and at follow-up. The patients were weighed in indoor clothing, without shoes. For five groups the follow-up session was digital due to Covid-19 restrictions and weight and height data was thus not collected. When height and weight data was not available, self-reported height and weight from the EDE-Q was used. Percent expected body weight (%EBW) was calculated as follows %EBW = BMI/50th percentile BMI for age and height × 100 [37].

#### Measures of remission

Recovery rates vary widely depending on the definition used [38]. We therefore report remission using several definitions.

*Psychological remission* was defined as EDE-Q global score below 2.5, which corresponds to the estimated cut-off score for EDE-Q in Norwegian samples [30].

Two measures of *weight remission* were used: (1) BMI percentile ≥ 25, which at the age of 15 (mean age of the current sample of adolescents) corresponds to a BMI of 18.5, the cut-off for underweight in World Health Organization guidelines [39], and (2) having achieved an EBW of 95% or greater. This criterion has been commonly used in research (e.g. [40, 41]) and has been shown to be an efficient predictor of long-term recovery for adolescent AN [42].

*Good, intermediate, and poor outcome* We also report remission according to the modified Morgan–Russel Global outcome scale [43, 44] that was used to report the outcomes of a large randomized multi-centre trial of MFT [20]. With these criteria, bulimic symptoms are taken into account, by using EDE-Q items 15, 16 and 17. Good outcome include adolescents whose weight is above 85% EBW, who are menstruating, and have no bulimic symptoms. Intermediate outcome include adolescents who meet the same weight criteria but are either not menstruating or having occasional bulimic symptoms (between one and three times over the past 28 days). Poor outcome is defined as having a weight below 85% EBW or having bulimic symptoms four times or more over the past 28 days. Male participants and those taking oral contraception (altogether 13% of total sample at start-of-treatment and follow-up, and 12% at end-of-treatment) were not included in the reports of this measure of remission. Contrary, adolescents reporting never to have menstruated (12% at start-of-treatment, 10% at end-of-treatment and 11% at follow-up) were included even though a quarter of them were below median age at menarche in Norway.

### Statistical analysis

The main outcome variables were investigated using linear mixed-effects modeling (LMM) to account for repeated measures by patient. Three separate models tested whether EDE-Q global score, SDQ total score, and BMI percentile changed from start-of-treatment to 6-month follow-up, and therefore included *Time* (start-of-treatment, end-of-treatment and follow-up) as a fixed effect. LMM has the advantage over repeated-measures ANOVAs that cases missing one or more observations are included in the analysis [45]. To investigate the effect of weight at start-of-treatment, *BMI percentile at start of treatment* was also included as a fixed effect. Interactions between *Time* and *BMI percentile at start-of-treatment* were checked for inclusion in the models. Variables with non-significant effects were not included in the final models. In the models for EDE-Q and SDQ, random intercept and slope for participant were included to account for differences between subjects in baseline scores and rate of change over time (i.e. relationship between the outcome variable and time). In the model for BMI percentile, only the random intercept was included. To examine the changes in the outcome variables during treatment and the following 6 months separately, we calculated estimated means for each time point and did pairwise comparisons of the estimated means.

The LMM analyses were performed using R [46] with the packages lme4 [47] and lmerTest [48]. The comparisons of estimated means were performed using the package emmeans in R. Effect sizes (ES) were calculated using the eff_size function in emmeans and can be interpreted using the same reference values as Cohen’s d: 0.2–0.49 is considered small, 0.50–0.79 is considered medium, and ≥ 0.8 is considered a large effect size [49]. To account for multiple testing, the level for statistical significance of all analyses was set to α = 0.01.

## Results

### Dropout

Of the 224 patients seen for MFT treatment at RASP between 2010 and 2021, 23 patients discontinued treatment (of these, 15 were among the 207 patients included in the present study), resulting in a dropout rate of 10.3%. Dropout was defined as attending fewer than 8 of 10 treatment days. For a few of the adolescents that discontinued treatment, the parents attended all the MFT meetings.

### Patient characteristics at start of MFT

The mean age at start-of-treatment was 15.0 ± 1.7 years (See Table 1). Self-reported mean age at first treatment for ED was 14.2 (± 2.1) years and self-reported mean duration of illness (i.e. time passed since the first time the patient experienced problems with weight or eating), was 1.8 (± 1.7) years. At start-of-treatment, the mean BMI of the adolescents was 17.6 ± 1.9 and mean BMI percentile 18.9 ± 19.7.

**Table 1:** Patient characteristics at start-of-treatment

		N
N total sample		207
Sex (% female)	91.3	207
Age		
Mean (years) ± SD	15.0 ± 1.7	207
Range	11–21	199
BMI		
Mean ± SD	17.6 ± 1.9	201
Range	12.8–24.6	201
BMI percentile		
Mean ± SD	18.9 ± 19.7	201
Range	0.01-96.0	201

### Changes in eating disorder symptomatology, psychological distress, and weight from start of treatment to six month follow-up.

#### Main models: effect of time and BMI at start-of-treatment on ED symptomatology, psychological distress and weight

**Fig. 1:**
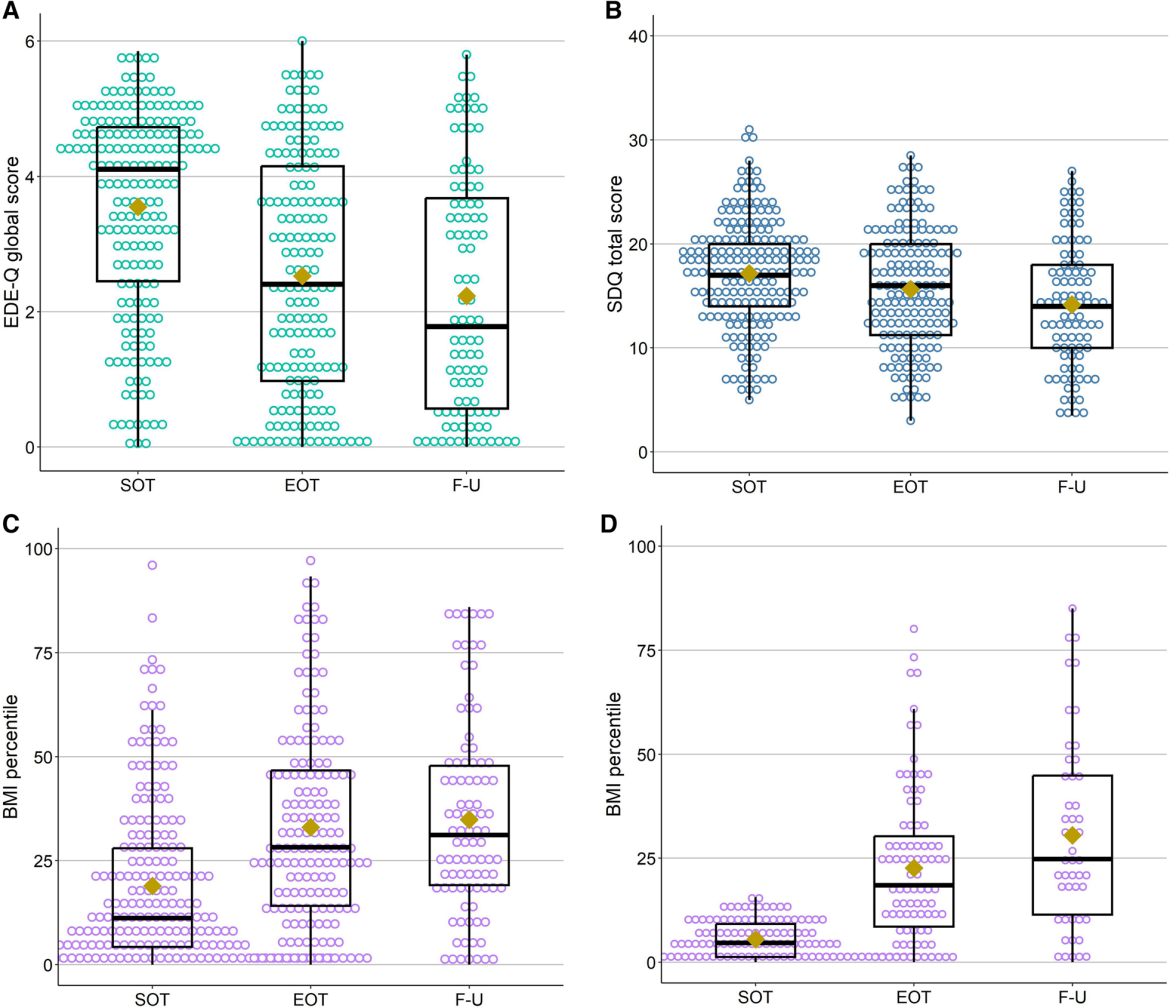
Mean and distribution of the three outcome variables at each time point.**A** EDE-Q Global score, **B** SDQ total score, **C** BMI percentile, full sample, **D** BMI percentile for only the subset of individuals who were underweight (i.e. BMI percentile < 16) at start-of-treatment. The yellow diamond represent mean values. Colored circles represent the data points of each individual patient. SOT, start-of-treatment; EOT, end-of-treatment; F-U, follow-up

 We used a linear mixed models approach to analyze the effect of time and BMI at start-of-treatment on ED symptomatology, psychological distress and weight. Statistically significant and non-significant fixed effects from the full model, and their coefficients, *p* values, and 95% confidence intervals, are presented in Table 2. See Fig. 1 for a visualization of the individual subject and mean change in EDE-Q, SDQ and BMI percentile from start-of-treatment to follow-up.

**Table 2:** Model parameters of the linear mixed model for EDE-Q, SDQ and BMI percentile

	Coef	95% CI	*p*
EDE-Q			
Intercept	3.3	3.02 to 3.60	< 0.001
Time	− 0.7	− 0.88 to − 0.57	< 0.001
BMI percentile _SOT_	0.0	− 0.00 to 0.02	0.157
SDQ			
Intercept	17.3	16.30 to 18.26	< 0.001
Time	− 1.6	− 2.04 to − 1.11	< 0.001
BMI percentile _SOT_	− 0.00	− 0.04 to 0.03	0.811
BMI percentile			
Intercept	0.9	− 1.83 to 3.69	0.511
Time	14.6	12.15 to 16.94	< 0.001
BMI percentile _SOT_	1.0	0.92 to 1.12	< 0.001
Time × BMI percentile _SOT_	− 0.3	− 0.41 to − 0.24	< 0.001

BMI percentile _SOT,_ BMI percentile at start-of-treatment; EDE-Q eating disorder examination questionnaire global score, SDQ strengths and difficulties questionnaire total score

In the model investigating EDE-Q global score, a significant effect of *Time* (− 0.7, 95% CI [− 0.88 to − 0.57], *p* < 0.001) indicates that EDE-Q decreased from start-of-treatment to 6 month follow-up, with on average 0.7 EDE-Q points per time period. The post hoc comparison of estimated means showed that the decrease in EDE-Q global score was significant during treatment (*p* < 0.001, ES = − 1.00), but not in the period from end-of-treatment to 6 month follow-up (*p* = 0.032, ES = 0.32) (Table 3). In the LMM, there was no significant effect of *BMI percentile at start-of-treatment* (0.0, 95% CI [0.00 to 0.02], *p* = 0.157, indicating that BMI percentile at start-of-treatment did not influence EDE-Q score.

**Table 3:** Comparison of estimated mean EDE-Q, SDQ and BMI percentile for the three time points

	M_1_	M_2_	Estimated diff	Effect size	95% CI	t (df)	*p*
EDE-Q							
SOT–EOT	3.5	2.5	− 1.0	1.00	1.24 to 0.77	8.5 (236)	< 0.001
EOT–FU	2.5	2.2	0.3	0.32	0.62 to 0.03	2.2 (247)	0.032
SOT–FU	3.5	2.2	1.3	1.32	1.64 to 1.01	8.4 (128)	< 0.001
SDQ							
SOT–EOT	17.2	15.7	− 1.45	− 0.51	− 2.13 to − 0.76	− 4.2 (234)	< 0.001
EOT–FU	15.7	14.0	− 1.75	− 0.61	− 2.64 to − 0.86	− 3.9 (243)	< 0.001
SOT–FU	17.2	14.0	− 3.20	− 1.12	− 4.17 to − 2.22	− 6.5 (128)	< 0.001
BMI percentile							
SOT–EOT	19.7	32.7	13.1	0.91	10.02 to 16.11	− 8.4 (290)	< 0.001
EOT–FU	32.7	33.6	0.9	0.06	2.95 to 4.66	− 0.4 (313)	0.659
SOT–FU	19.7	33.6	13.9	0.97	10.24 to 17.59	− 7.4 (331)	< 0.001

*EDE-Q* eating disorder examination questionnaire global score, *EOT* end-of-treatment, *FU* follow-up, *SDQ* strengths and difficulties questionnaire total score, *SOT* start-of-treatment

For BMI percentile, the pairwise comparisons were based on a model without the interaction term included in the LMM

In the model investigating SDQ total score, a significant effect of *Time* (− 1.6, 95% CI [− 2.04 to − 1.11], *p* < 0.001) indicates that SDQ total score decreased over time, with on average 1.6 SDQ points per time period. There was no significant effect of *BMI percentile at start-of-treatment* on SDQ total score (0.0, 95% CI [− 0.04 to 0.03], *p* = 0.811). Comparison of estimated means showed that there was a significant difference in SDQ between both start-of-treatment and end-of-treatment (*p* < 0.001, ES = − 0.51), and end-of-treatment and follow-up (*p* < 0.001, ES = − 0.61), with medium effect sizes for both time periods.

In the model investigating BMI percentile, a significant effect of *Time* (14.6, 95% CI [47.42 to 79.83], *p* < 0.001) indicates that on average the BMI percentile increased with 14.6 from start-of-treatment to 6 month follow-up. A significant effect of *BMI percentile at start-of-treatment* (1.0, 95% CI [0.92 to 1.12], *p* < 0.001) indicates that higher BMI at start-of-treatment was associated with higher mean BMI percentile across the three time points. A significant interaction between *Time* and *BMI percentile at start-of-treatment* (− 0.3, 95% CI [− 0.41 to -0.24], *p* < 0.001) indicates that lower BMI at start-of-treatment was associated with larger increase in BMI percentile from start-of-treatment to follow-up. Pairwise comparisons of estimated means at the three time points showed that there was a significant change in BMI percentile during treatment (*p* < 0.001, ES = 0.89), but not from end-of-treatment to the 6 month follow-up ((*p* = 0.365, ES = 0.09) (See Table 3). Note that the pairwise comparisons were based on a model without the interaction term and does not take into account BMI percentile at start-of-treatment.

#### Post hoc investigation of change in BMI percentile in the adolescents who were underweight at start-of-treatment

A post hoc LMM, with *Time* and *BMI percentile at start-of-treatment* as fixed effects, was performed to investigate the change in BMI percentile over time in the subsample of adolescents with a BMI percentile below 16 at start-of-treatment (n = 116). Like in the model for the full sample, there were significant effects of *Time* (12.7, 95% CI [10.55 to 14.93], *p* < 0.001) and *BMI percentile at start-of-treatment* (1.0, 95% CI [0.55 to 1.45], *p* < 0.001). Contrary, the interaction between Time and BMI percentile at start-of-treatment was not significant for the underweight adolescents. Moreover, comparisons of estimated means for the underweight adolescents showed that for this subsample, the increase in BMI percentile was significant not only from start-of-treatment to follow-up, but also from end-of-treatment to the 6 month follow-up (See Table 4).

**Table 4:** Change in BMI persentile for adolescents being underweight at start-of-treatment

	Coef	95% CI	*p*
Linear mixed model			
Intercept	1.1	− 2.47 to -4.58	0.558
Time	12.7	10.55 to 14.93	< 0.001
BMI percentile _SOT_	1.0	0.55 to 1.45	< 0.001

	M_1_	M_2_	Est. diff	Effect size	95% CI	t (df)	*p*
Comparison of estimated means							
SOT–EOT	5.8	22.5	16.7	1.32	13.1 to 20.4	9.1 (162)	< 0.001
EOT–FU	22.5	29.5	7.1	0.56	2.4 to 11.7	3.0 (172)	0.003
SOT–FU	5.8	29.5	23.8	1.88	19.3 to 28.3	10.4 (184)	< 0.001

*BMI percentile*_*SOT*_ BMI percentile at start-of-treatment, *EOT* end-of-treatment, * FU* follow-up, *LMM* linear mixed model, *SOT* start-of-treatment

#### Duration of treatment: post hoc comparison of within-treatment change between adolescents receiving 10 versus 5 months of MFT treatment

We performed a post hoc LMM analysis with *time* and *group* (5 vs. 10 months treatment duration) as fixed factors to explore whether there was a difference in within-treatment change between adolescents who received MFT over 10 months versus adolescents who received MFT over 5 month. There were no significant effects of *Group* (*p* values: EDEQ: 0.647; SDQ: 0.611; BMI percentile: 0.084) and no significant interactions between *Group* and *Time* (*p* values: EDEQ: 0.392; SDQ: 0.0137; BMI percentile: 0.257).

### Categorized clinical outcome at start-of-treatment, end of treatment and follow-up

At end-of-treatment 43.2% of the adolescents had reached weight remission according to the definition of weight remission as %EBW of 95 or more, and 59.5% had achieved weight remission according to the definition of full weight remission as having a BMI percentile equal to or more than 25 (See Table 5). At follow-up, these numbers had increased to 49.3% and 60.9%, respectively. Psychological remission, defined by a global EDE-Q score equal to or below 2.5, was reached by 50.9% of the adolescents at end-of-treatment and 56.3% at follow-up. For comparison, at start-of-treatment only 25.8% had an EDE-Q score of ≤ 2.5.

**Table 5:** Number of adolescents in remission as defined by different criteria

	Valid % (frequency)		
	Start-of-treatment	End-of treatment	Follow-up
Psychological remission			
EDE-Q global score ≤ 2.5	25.8 (49 of 190)	50.9 (83 of 163)	56.3 (54 of 96)
Weight remission			
BMI percentile ≥ 25	28.4 (57 of 201)	59.5 (94 of 158)	60.9 (56 of 92)
% EBW ≥ 95	19.8 (38 of 192)	43.2 (67 of 155)	49.3 (37 of 75)
Global outcome (M–R)			
Good	12.6 (16 of 127)	30.4 (34 of 112)	47.3 (26 of 55)
Intermediate	35.4 (45 of 127)	40.2 (45 of 112)	29.1 (16 of 55)

*EBW* expected body weight, *EDE-Q* eating disorder examination questionnaire global score, *M–R* Morgan–Russel

**Fig. 2:**
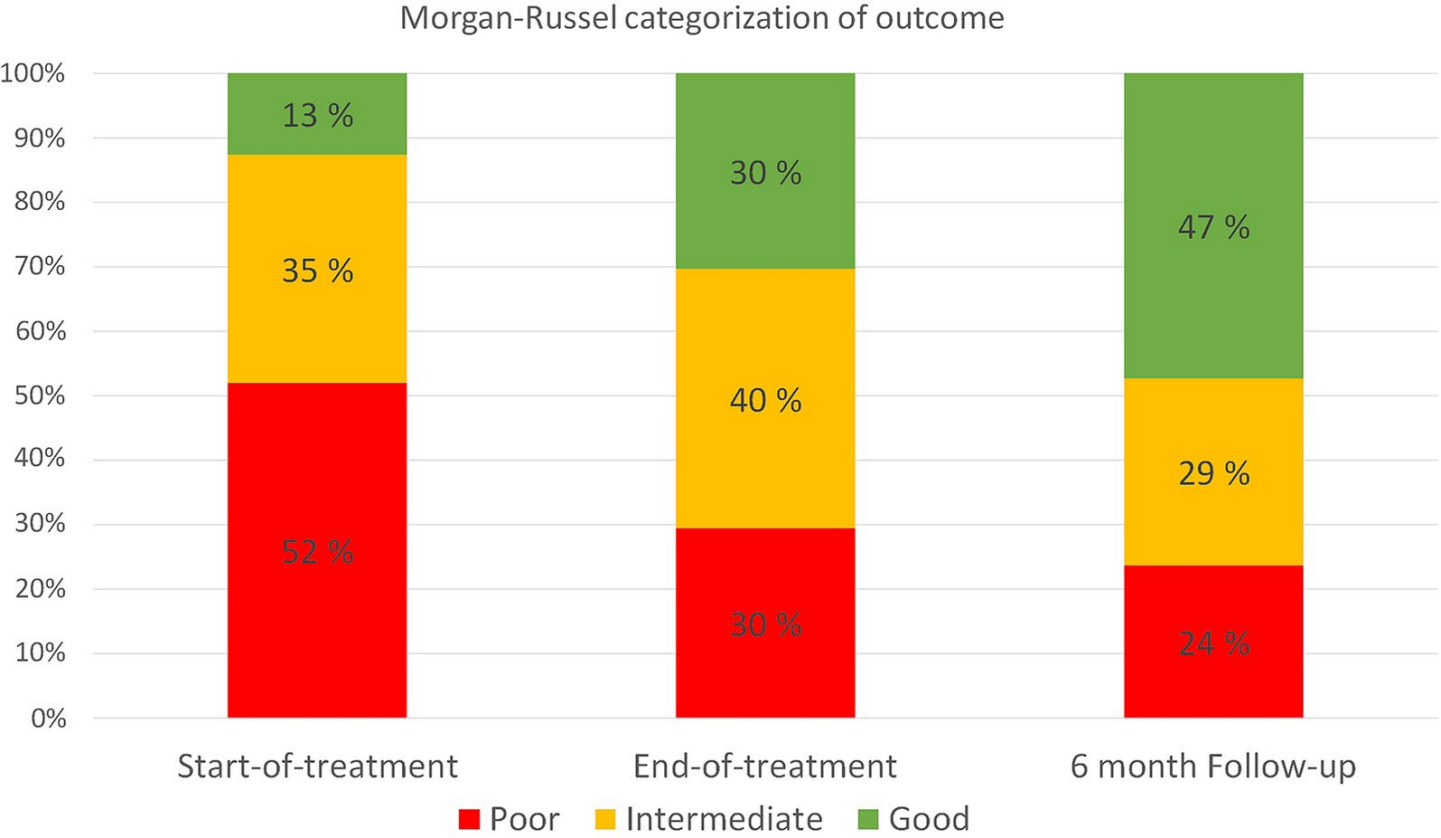
Distribution of Morgan–Russel categorization at start of treatment, end of treatment and follow-up. Note: Male participants and those taking oral contraception are not included in the calculation of the outcome measures presented in this figure. The number of boys/girls taking oral contraception were 18/9 at start-of-treatment, 7/18 at end-of-treatment, and 7/12 at follow-up

According to the Morgan–Russel scale, 70.6% (30.4% good, 40.2% intermediate) of the adolescents had a good or intermediate outcome status at end-of-treatment, and 76.4% (47.3% good, 29.1% intermediate) achieved the same status at follow-up (not including male participants and those taking oral contraception) (See Fig. 2). For the subsample of adolescents with underweight at start-of-treatment the percentages were as follows: Start-of-treatment: 3.8% good, 25.3% intermediate, 70.9% poor; End-of-treatment: 32.8% good, 37.5% intermediate, 29.7% poor; Follow-up: 44.8% good, 27.6% intermediate, 27.6% poor.

## Discussion

This naturalistic study on 207 adolescents who participated in MFT aimed to evaluate the outcome of outpatient MFT in a routine clinical setting. There were significant improvements in the adolescents’ ED symptomatology, psychological distress and weight from start-of-treatment to 6 month follow-up. Acceptability of the treatment seemed high, as only 10% discontinued treatment. The low dropout is in line with previous MFT studies, as reported in a recent meta-analysis [50].

### Changes in eating disorder symptomatology

There was a significant reduction in global EDE-Q score during treatment, and by follow-up, mean EDE-Q global score was below the clinical cut-off score [30]. The mean BMI percentile of the full sample was above the cutoff for a healthy weight already at start-of treatment (BMI percentile > 16). Largely, this was due to many patients diagnosed with F50.0 AN at referral, no longer meeting the weight criterion for this diagnosis at start of MFT. They had reduced the most severe underweight during ED treatment at local specialized mental health services prior to start of MFT. Additionally, our sample included a few patients with diagnoses other than AN. For the subsample of adolescents with a BMI percentile below 16 at start-of-treatment, mean BMI percentile was well below the cutoff for a healthy weight at start-of-treatment (estimated mean BMI percentile at start-of-treatment was 5.8). By end of treatment, the mean BMI percentile of this subgroup had increased to 22.5, which is well above the cutoff for a healthy weight.

The largest improvements in ED psychopathology and BMI percentile were obtained during treatment. The effect sizes of these changes were large. This is comparable to, or better than, previous studies [50]. Improvements in both ED psychopathology and weight suggest that MFT is an effective treatment for eating disorders. The lack of control group in several studies (e.g. [51–55]), including the current study, calls for caution in interpreting the unique effects of MFT. However, a large RCT showing that adjunct MFT improved the outcomes of single-family therapy (FT-AN) [20], suggests that MFT adds to the effectiveness of single-family therapy.

### Did weight at start of treatment affect the results?

For the full sample, weight at start-of-treatment affected the rate of increase in BMI percentile over time. The lower the BMI at start-of-treatment, the larger was the change in BMI percentile from start-of-treatment to follow-up. This presumably reflects that underweight adolescents, who needed to gain weight, did indeed gain weight, while adolescents at a healthy weight, who were rather in need of weight stabilization, did not increase their weight. The level of ED symptomatology and psychological distress, on the other hand, and the rate of change over time in these variables, did not differ as a function of weight at start-of-treatment. These results indicate that MFT can be a valuable treatment for not just a subset of patients, but rather for adolescents with differing ED related challenges and weight ranges.

### Changes in psychological distress

The adolescents in the current study experienced a significant decrease in self-rated psychological distress across treatment and the following 6 months. At start-of-treatment, mean SDQ total score was below the proposed Norwegian cut-off [35], but within the borderline range between an abnormal and healthy score. By follow-up, the mean score was within healthy range. To our knowledge, no study has previously reported on the change in SDQ scores from start to end of MFT, but a few studies have investigated general psychological well-being. A recent meta-analysis on MFT for EDs identified only three studies that reported pre-post data on patients’ general psychological well-being [50]. The three studies were all on adolescents. Dennhag et al. (2021) found that adolescents significantly improved in global function pre- to post-MFT [51]. The other two studies assessed quality of life [52, 56]. Both studies reported significant improvements in quality of life from before to after MFT. The meta-analysis found that overall, the three studies showed a significant increase in patients’ general psychological well-being, with a large effect size. This is comparable to what was found in the present study, where the change in SDQ total score from start-of-treatment of end-of-treatment had a medium effect size, and the change from start-of-treatment to 6 month follow-up had a large effect size. Together these studies suggest that MFT not only improves ED symptomatology, but can also improve general psychological well-being, including decreasing psychological distress.

### Change during treatment versus after treatment

In the current study, psychological distress decreased significantly during as well as after treatment. For EDE-Q and BMI percentile, the largest changes were obtained during treatment, although, for the underweight subsample, the change in BMI percentile was significant at both time periods. Our findings are in line with the results of the few previous studies investigating changes occurring during and after outpatient MFT. I.e. that improvements in ED psychopathology, weight and emotion regulation achieved during treatment are generally maintained or improved at follow-up [20, 22, 53, 57].

The continued improvements after MFT treatment could be after-effects of treatment. Adolescents and their families continue the behavioral and psychological changes started through MFT and continue to apply the techniques learnt in MFT. In line with this, Couturier et al. (2013) explained the difference they found between the efficacy of FBT and individual therapy at follow-up, but not at end-of-treatment, by the fact that those who had underwent FBT still have the support of their parents after treatment [58]. The parents have learnt techniques in FBT that they can continue to apply long after end of treatment. The continued reductions in psychological distress could also reflect that the illness gradually takes up less space in daily life due to the improvements made during MFT, and that this, in turn, reduces general psychological distress. Although these factors might be possible reasons for continued improvement after end of MFT, in the present study we cannot rule out the possibility that the continued improvements are rather or in large part effects of continued ED treatment, as most of the adolescents continued in treatment for their ED after the end of MFT.

### Categorical outcomes

To allow for comparison with the remission rates of the only RCT on outpatient MFT for adolescents [20], we used the Morgan–Russel Global outcome scale. Our numbers were comparable to the RCT, with the percentage having a good outcome at 6 month follow-up being approximately the same. At end-of-treatment, the percentage of adolescents having a good or intermediate outcome was only slightly lower in our sample (70%) than in the RCT (76%), despite the fact that for 2/3 of our sample, MFT treatment lasted for a shorter period, leaving shorter time for changes to take place. This shows that MFT performs equally well in a real world clinical setting as in a research setting. In another study that used the Morgan–Russel scale, 62% of the adolescents were in the good or intermediate outcome groups 6 months into a 9-months MFT [55].

Important to note is that in the present study, 13% of the adolescents were classified as belonging to the “good outcome” group already at start-of-treatment, as compared to 0% in the RCT and the study by Salaminiou et al. (2017). This could indicate that our sample included less severe cases of EDs than in the other two studies. However, we believe that this is not the case. Patients receiving MFT at RASP are referred from a large number of specialized mental health services. Only a small portion of the ED patients at these services are referred for MFT, and it is likely that this is the proportion of patients most in need of more help and/or that has been ill for the longest time. As previously mentioned, many patients that had an F50.0 AN diagnosis at the time they were referred, reduced their severe underweight in treatment at the local specialized mental health services before start of MFT. When the outcome numbers were calculated for the adolescents who were underweight at start-of-treatment, the percentage of adolescents classified as belonging to the “good outcome” group at start-of-treatment, decreased to 3.8%, while the outcomes at end-of-treatment and follow-up remained approximately the same as for the full sample.

Taken together, the current study supports the findings of the few previous studies reporting categorical outcomes after MFT. I.e. more than 60% achieve an intermediate or good outcome as classified using a modified Morgan–Russel scale or similar criteria at or close to end-of-treatment, and that at 6 months follow-up, the numbers are relatively unchanged. Importantly, this study also shows that MFT perform equally well in an everyday specialist clinical setting as in a research setting.

### Clinical implications

This study showed that MFT performs equally well in a real world specialist clinical setting as in a research setting. Together with the promising outcome numbers of MFT, this suggests that MFT could be a valuable treatment for many clinics treating children and adolescents with EDs. However, it should be noted that running MFT groups requires a lot of time and resources, and having a dedicated treatment team is an advantage to ensure stability, predictability and quality. The MFT team at RASP consists of highly experienced clinicians with dedicated time focused on managing and delivering MFT. Hence, the promising outcome numbers in the current study could be partly attributed to the experience of the clinical team.

While this and previous MFT studies add to a growing literature showing the utility of the MFT approach for adolescents with an eating disorder, the study also adds to the literature showing that a large proportion are still not fully recovered at end of MFT treatment or at follow-up. This is also true for other ED treatments. For example, about 50% of patients treated with FBT experience remission [59, 61]. With 60% or more achieving a good or intermediate outcome after MFT, MFT shows comparable numbers to the outcome numbers for FBT, although caution needs to be taken when comparing outcome across studies. Recovery rates vary greatly depending on the definition used [38, 61]. In the present study, the patients show continued improvements in the 6 month follow-up period, and more patients have a good outcome at follow-up compared to at end-of-treatment. Although this might be partly attributed to continued ED treatment, it brings hope that many continue to improve also after completing MFT. Future studies should investigate what subsets of patients benefit the most from MFT, and more large RCT’s are needed to assess what MFT offers in addition to single-family therapy.

### Strengths and limitations

Being an uncontrolled naturalistic study where data was collected through clinical audit, the study does not have a comparison treatment or other control group. Moreover, we do not have reliable data on the type and amount of adjunct treatment the participants received during or after MFT. The unique contributions of MFT to the observed improvements can thus not be ascertained. Also due to the naturalistic nature of the study, treatment duration changed during the time of data collection. Moreover, data were collected at follow-up from only about 2/3 of the patients.

## Conclusion

With a study sample of more than 200 adolescents, the present study is the largest study on MFT for EDs to date. We found that adolescents treated with adjunct outpatient MFT for eating disorders experience significant improvements in ED symptomatology, psychological distress and weight from start-of-treatment to end-of-treatment and to 6 month follow-up. This contribution supports findings from smaller uncontrolled studies and one RCT showing the utility of the multi-family approach. Showing comparable remission rates to an RCT, this study importantly shows that outcomes in a routine clinical practice compare to those reported in a research trial.

## Data Availability

The study does not have ethical approval to share data.

## Abbreviations

AN: Anorexia nervosa
BMI: Body mass index
BN: Bulimia nervosa
ED: Eating disorder
EDE-Q: Eating disorder examination questionnaire
FBT: Family-based treatment
FT–AN: Family therapy for eating disorders/Maudsley model of family therapy
LMM: Linear mixed-effects modeling
MFT: Multifamily therapy
RASP: Regional Department for Eating Disorders
SDQ: Strengths and difficulties questionnaire
